# Solid-State NMR Spectra of Protons and Quadrupolar Nuclei at 28.2 T: Resolving Signatures of Surface Sites with Fast Magic Angle Spinning

**DOI:** 10.1021/jacsau.2c00510

**Published:** 2022-10-25

**Authors:** Zachariah J. Berkson, Snædís Björgvinsdóttir, Alexander Yakimov, Domenico Gioffrè, Maciej D. Korzyński, Alexander B. Barnes, Christophe Copéret

**Affiliations:** † Department of Chemistry and Applied Biosciences, ETH Zürich, Vladimir Prelog Weg 2, Zürich 8093, Switzerland

**Keywords:** solid-state NMR spectroscopy, high-field, surface sites, catalysts

## Abstract

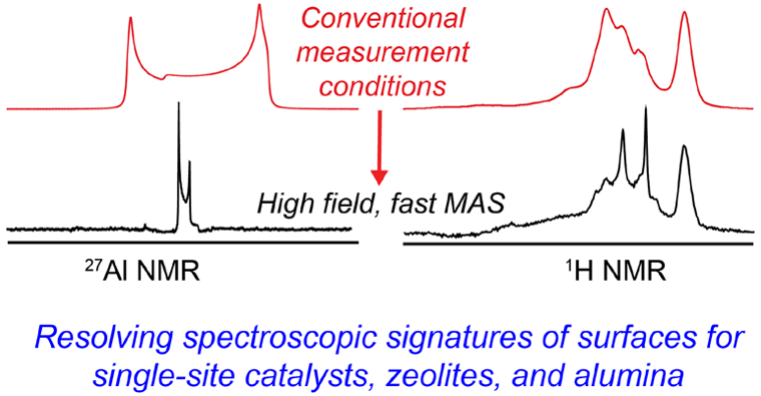

Advances in solid-state nuclear magnetic resonance (NMR) methods and hardware offer expanding opportunities for analysis of materials, interfaces, and surfaces. Here, we demonstrate the application of a very high magnetic field strength of 28.2 T and fast magic-angle-spinning rates (MAS, >40 kHz) to surface species relevant to catalysis. Specifically, we present as case studies the 1D and 2D solid-state NMR spectra of important catalyst and support materials, ranging from a well-defined silica-supported organometallic catalyst to dehydroxylated γ-alumina and zeolite solid acids. The high field and fast-MAS measurement conditions substantially improve spectral resolution and narrow NMR signals, which is particularly beneficial for solid-state 1D and 2D NMR analysis of ^1^H and quadrupolar nuclei such as ^27^Al at surfaces.

Solid-state nuclear magnetic resonance (NMR) is a powerful tool for materials characterization, with applications spanning biomolecules,^1^ polymers,^2^ battery materials,^3^ semiconductors,^4^ and catalysts.^5^ It can provide precise element-specific information on the local structure, interactions, and dynamics of NMR active nuclei.^6^ However, it is limited by its intrinsically low sensitivity due to low nuclear spin polarization and by signal broadening due largely to strong internuclear and/or quadrupolar interactions and inhomogeneous distributions of chemical species that yield corresponding distributions of chemical shifts.

Measurements at increasingly high magnetic field strengths improve both signal sensitivity and spectral resolution. The ongoing development of NMR instrumentation including stable high magnetic fields >20 T and fast-spinning NMR probeheads capable of MAS rates up to 150 kHz has enabled new opportunities for understanding biomolecules^7−9^ and determining their 3D structures in the solid-state^10^ including challenging cases such as metalloproteins^11^ and membrane proteins in native environments.^12^ However, the application of these capabilities to functional inorganic materials and their surfaces, including catalysts, has been so far more limited. In fact, very high magnetic fields and fast MAS rates would be especially powerful to characterize such materials,^13,14^ in particular for the analysis of surface sites that are associated with highly unsymmetrical inhomogeneous environments with broad and complex spectroscopic signatures that are challenging to measure and interpret under typical conditions. NMR analysis of quadrupolar nuclei, for example, greatly benefits from very high magnetic fields,^15−18^ as demonstrated by recent studies of ^27^Al,^19−21^^17^O,^22,23^^67^Zn,^24^ and ^95^Mo nuclei^25^ in materials like aluminosilicate zeolites, alumina, and metal organic frameworks at magnetic fields up to 36 T and MAS rates up to 30 kHz. Despite the inhomogeneously broadened lineshapes, NMR of such materials can also benefit from very fast MAS rates (>40 kHz), though applications have been limited to selected cases such as organic–inorganic hybrid materials^26^ and resolving paramagnetic shifts in inorganic oxides.^27,28^

With commercial NMR spectrometers now operating at 28.2 T (1200 MHz for ^1^H), we became interested in exploiting these high stable magnetic fields combined with fast-spinning solid-state NMR probes for analysis of inorganic oxides, particularly focusing on the structures and dynamics of surface species relevant to catalysis. Here, we highlight several representative case studies showing the dramatic improvements in resolution that can be obtained for inorganic systems. This is demonstrated for a well-defined silica-supported organometallic catalyst, dehydroxylated γ-alumina and a zeolite as illustrative examples. The improved signal resolution, particularly for proton NMR and quadrupolar nuclei, yields highly resolved 1D and 2D MAS NMR spectra that contain key information on surface structures, validating the approach.

We focus first on assessing the resolution obtained in ^1^H MAS NMR spectra of heterogeneous materials, which can be significantly broadened due to inhomogeneous effects like chemical shift dispersion.^18^ Nevertheless, the resolution benefits of fast MAS and 28.2 T acquisition conditions are still remarkable. This is illustrated by the 1D ^1^H MAS NMR spectra of a well-defined silica-supported organometallic species,^29,30^ the W alkylidene (ArN)W(Me_2_Pyr)_2_ (CHCMe_2_Ph) (Ar = 3,5-dimethyl-phenyl; Me_2_Pyr = 1,4-dimethylpyrrolide) grafted on partially dehydroxylated silica (SI Figure S2.1). Signals from different methyl, pyrrolidine, aromatic, and alkylidene ^1^H species are broad and unresolved under conventional measurement conditions (Figure 1, red). Resolution improves considerably at 28.2 T and 65 kHz MAS (Figure 1, blue, black; SI Section S2). Notably, the ^1^H NMR signal of the alkylidene proton is well-separated from the aromatic resonances. A shoulder at 10.5 ppm is also partially resolved and assigned based on literature reports to the *anti* alkylidene rotamer,^31^ typically present in lower amounts compared to the *syn* rotamer, which shows a more intense signal at 9.4 ppm. The resolution of different alkylidene species by ^1^H NMR in the solid state is noteworthy, as the alkylidene moiety is responsible for their olefin metathesis catalytic activity and is typically impossible to observe by ^13^C MAS NMR without isotopic enrichment.^32,33^ Such highly resolved ^1^H MAS NMR spectra could support and improve NMR-based tools for three-dimensional structural determination of surface species.^34,35^

**Figure 1:**
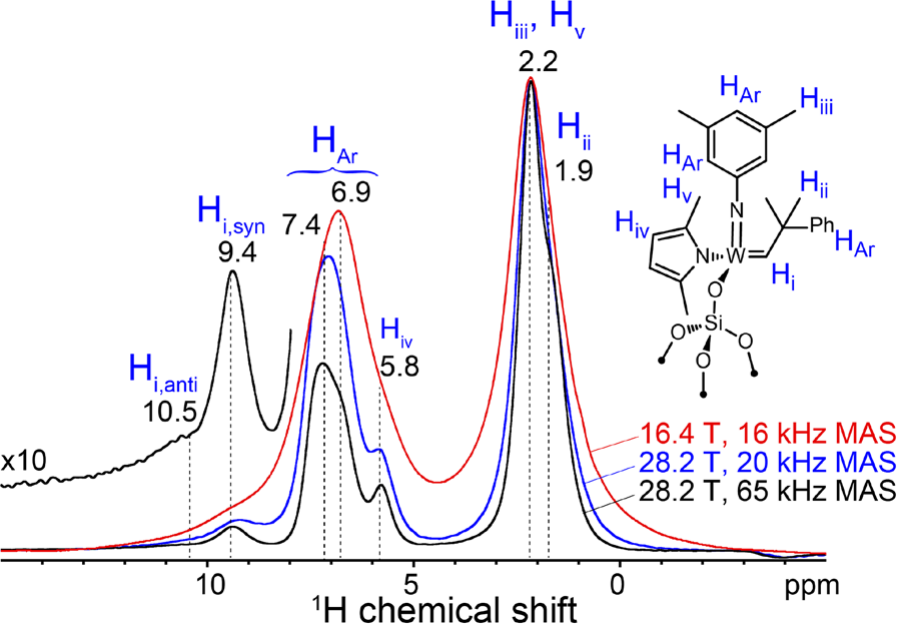
Solid-state 1D ^1^H echo MAS NMR spectra of a silica-supported W alkylidene (inset) acquired at 285 K, 16.4 T, and 16 kHz MAS (red) or 28.2 T and 20 kHz MAS (blue) or 65 kHz MAS (black). Signal assignments are indicated on the schematic inset.

As a second case study, we observe substantially improved resolution in the solid-state ^1^H MAS NMR spectra of needle-shaped γ-alumina as a function of magnetic field and MAS rate. Recently, we reported the synthesis and solid-state NMR characterization of needle-shaped γ-alumina crystallites with a larger proportion of edge and surface sites.^36^ The ^1^H MAS NMR spectra of the γ-alumina needles resolve surprisingly narrow ^1^H signals from different OH sites, with resolution improving with both higher field and faster MAS (Figure 2a,b). At 28.2 T and 50 kHz MAS, five ^1^H NMR signals are clearly resolved at −0.1, 1.1, 1.7, 2.2, and 2.5 ppm, as well as a shoulder at 3.5 ppm. Based on recent experimental and computational analyses,^36,37^ the signal at −0.1 ppm is assigned to μ_1_ Al–OH moieties, those in the 1.1–2.5 ppm region to bridging μ_2_ Al–OH–Al moieties, and the shoulder at 3.5 ppm to H-bond donors. To our knowledge, such complex and well-resolved ^1^H NMR signals have not previously been observed in ^1^H MAS NMR spectra of γ-alumina. These results open the possibility of identifying signals from Al–OH species at specific edge and facet sites, linking their local structures and corresponding reactivities. Toward this goal, analyses of the present results show that distinct μ_2_ Al–OH species participate to different extents in a network of interacting and dipole–dipole coupled surface OH groups. Only the signals at 1.1 and 1.7 ppm narrow substantially with increasing MAS rate (Figure 2a,b, Figure S3.1). The different MAS-dependencies of the ^1^H signals suggest the influence of substantial ^1^H dipole–dipole couplings for specific surface ^1^H species and appear related to their very different measured nuclear spin relaxation time behavior (Table S3.1). The mutual interactions of these specific sites are corroborated by 2D ^1^H{^1^H} nuclear Overhauser effect spectra (NOESY, Figure S3.2), which show that the bridging μ_2_–OH species associated with the ^1^H signals at 1.1 and 1.7 ppm are highly dynamic/fluctional and are in very close mutual spatial proximity compared to those with ^1^H signals at 2.2 and 2.5 ppm. These physiochemical insights can provide valuable constraints on models of the γ-alumina surface, the structure of which is still a matter of considerable investigation.

**Figure 2:**
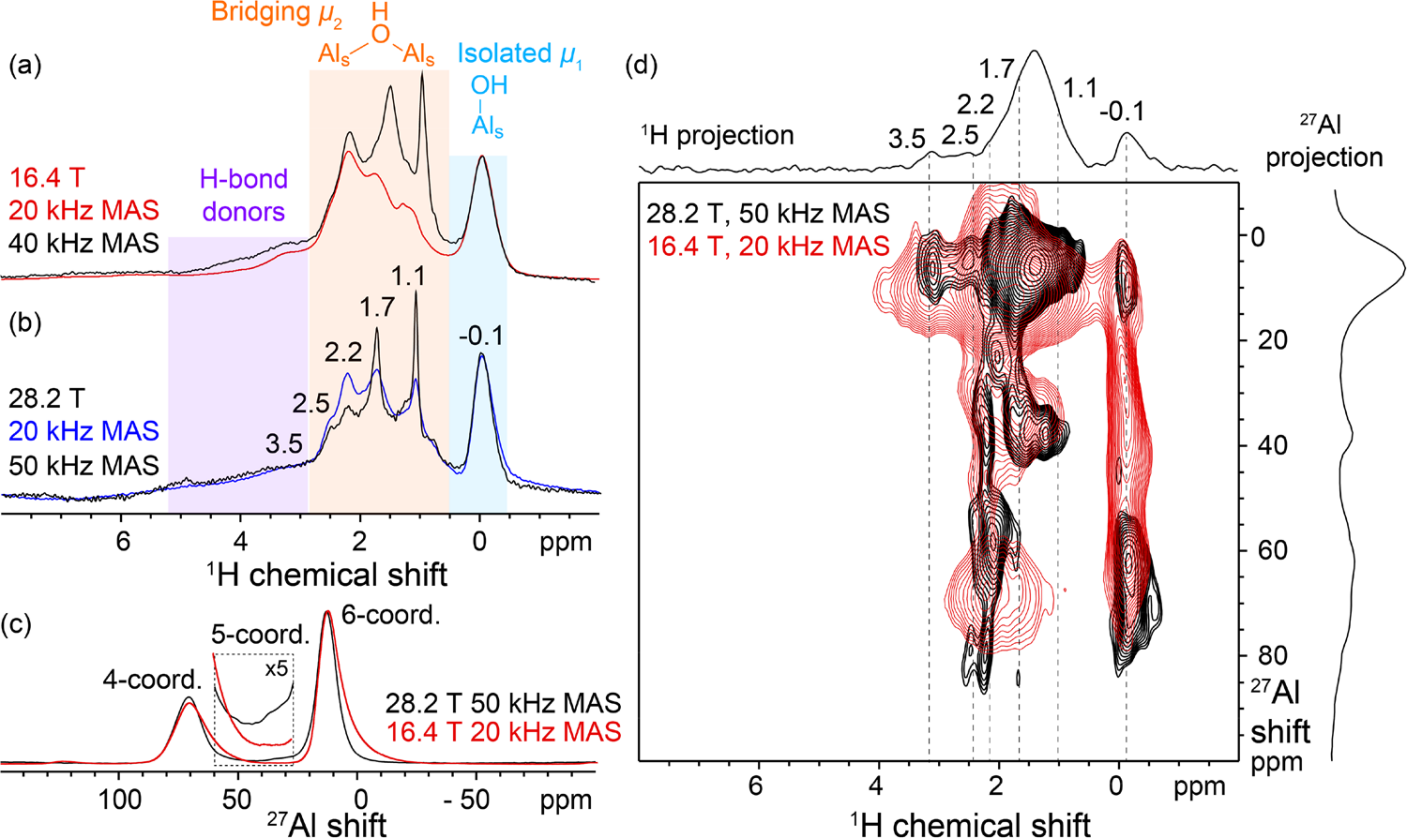
Solid-state NMR spectra of needle-shaped γ-alumina crystallites: 1D ^1^H echo MAS NMR spectra acquired at (a) 16.4 T and 20 kHz MAS (red) or 40 kHz MAS (black) or (b) 28.2 T and 20 kHz (blue) or 50 kHz MAS (black). (c) 1D single pulse ^27^Al MAS NMR spectra acquired at 16.4 T and 20 kHz MAS (red) or 28.2 T and 50 kHz MAS (black). (d) Overlay of 2D ^1^H{^27^Al} AID-D-HMQC correlation spectra acquired at 16.4 T and 20 kHz MAS (red) or 28.2 T and 50 kHz MAS (black).

The second-order quadrupolar contribution to the NMR lineshapes of quadrupolar nuclei like ^27^Al depends inversely on magnetic field strength,^15^ yielding narrower lines at higher fields. For the needle-shaped γ-alumina crystallites, this effect substantially narrows the ^27^Al signals from 4- and 6-coordinate Al sites in the bulk of the material (Figure 2c) and reveals a weak ^27^Al signal at 35 ppm from 5-coordinate Al surface sites, comprising ca. 2% of the total Al (Figure S3.3).^21^ Along with the narrowing of ^1^H resonances with increasing MAS rate, this yields remarkable improvement in both ^27^Al and ^1^H dimensions of 2D ^1^H{^27^Al} arbitrary indirect dwell (AID)^38^ dipolar heteronuclear multiple quantum coherence (D-HMQC) correlation spectra (Figure 2d) compared to spectra acquired at 16.4 T and 20 kHz. The fast MAS conditions also allow shorter rotor-synchronized dipolar recoupling periods (12 rotor periods for recoupling equates to 0.24 ms at 50 kHz MAS compared to 0.6 ms at 20 kHz MAS). As a result, shorter-range and stronger ^1^H–^27^Al interactions may be accessed. The ^1^H signal at ca. −0.1 ppm was previously observed to correlate with ^27^Al signals having quadrupolar coupling constant (*C*_Q_) values of 8–13.5 MHz in similar 2D D-HMQC spectra acquired at 16.4 T.^36^ However, this value is lower than expected for tetrahedrally coordinated ^27^Al sites on the surface of highly dehydroxylated γ-alumina based on first-principles calculations.^39^ It has been suggested that such ^1^H–^27^Al double-resonance experiments might enhance relatively narrow signals from subsurface ^27^Al sites, rather than broader signals from surface species.^39^ Correspondingly, unambiguous insights into the nature of γ-alumina surfaces have been elusive. Lineshape analyses of 1D slices of the 2D ^1^H{^27^Al} D-HMQC spectrum indicate the presence of correlated ^27^Al signals with *C*_Q_ values of at least 15.5 MHz (Figure S3.4, Table S3.2), within the 15–20 MHz range calculated for tetrahedrally coordinated surface ^27^Al sites. The combination of very high magnetic fields and fast spinning thus appears a promising route to detect surface species.

The extensibility of the high field and fast MAS conditions to diverse material systems is illustrated by analysis of a prototypical aluminosilicate catalyst, dehydrated microporous mordenite zeolite. Elucidating the local structures of aluminum heteroatoms in zeolites is of great importance in understanding their reactivities, though the nature and distributions of framework and extra-framework Al sites have long been elusive.^40^ This is particularly true after dehydration of the framework, which leads to significant broadening of ^27^Al NMR signals. Recently, our group provided evidence that the Lewis acid sites in mordenite zeolite are pseudo-tricoordinate framework Al interacting with a coordinated siloxane bridge.^41^ The 1D ^27^Al NMR spectrum of dehydrated mordenite is substantially narrowed at 28.2 T compared to 16.4 T (Figure 3a), and two different signals are resolved that can be associated by analysis of the 2D ^27^Al{^1^H} D-HMQC spectra with Bro̷nsted and Lewis acid sites (Figure 3b) as previously discussed.^41^ The 2D ^27^Al triple-quantum MAS (TQMAS) spectrum^42^ of the zeolite (Figure 3c) separates the two signals further and enables their spectroscopic parameters to be estimated, yielding *C*_Q_ values consistent with those previously reported.^41^

**Figure 3:**
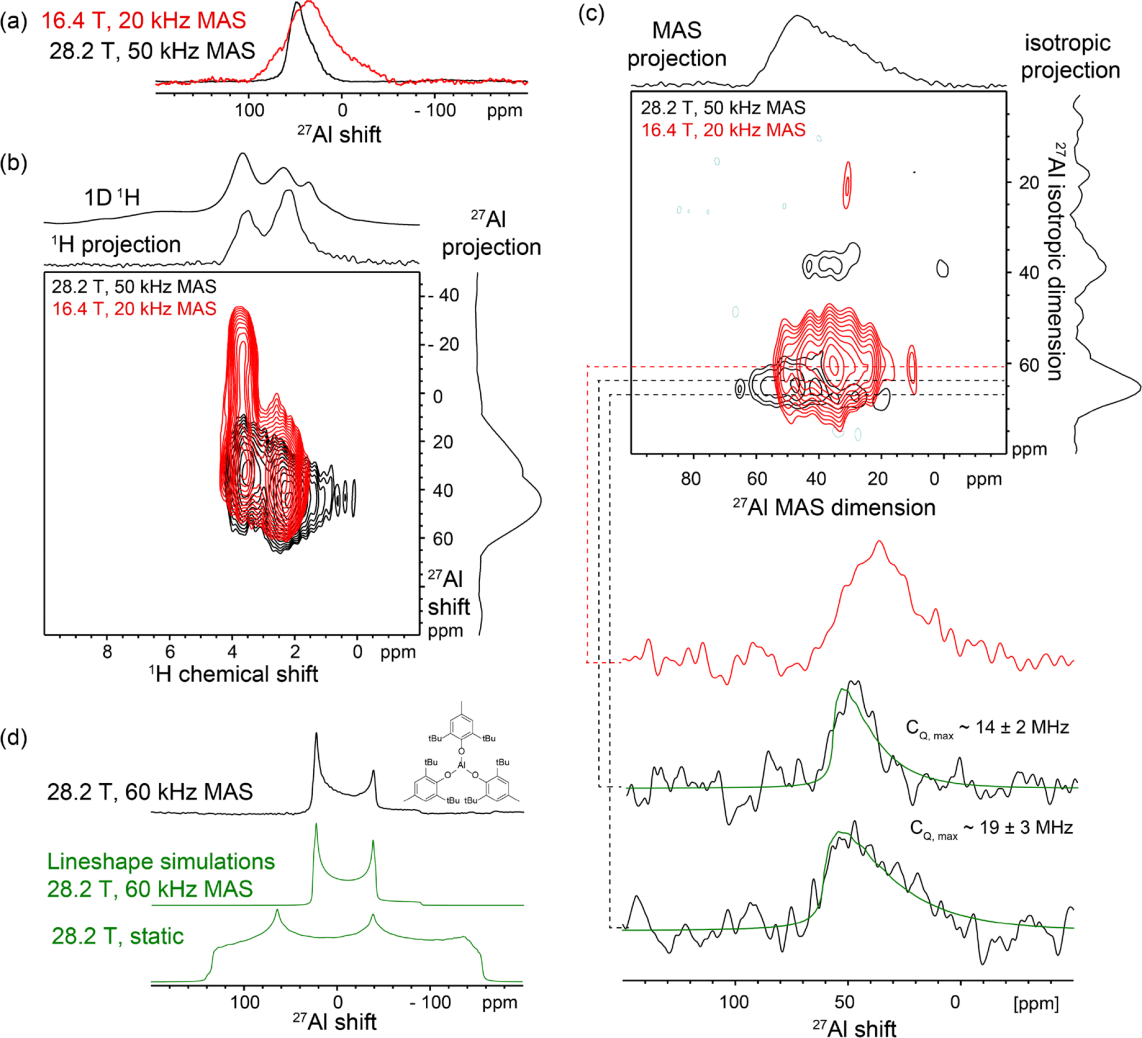
Solid-state (a) 1D ^27^Al MAS, (b) 2D ^1^H{^27^Al} AID-HMQC, or (c) 2D ^27^Al TQMAS NMR spectra of dehydrated mordenite zeolite acquired at (red) 16.4 T and 20 kHz MAS or (black) 28.2 T and 50 kHz MAS. Slices extracted from the 2D TQMAS spectra at positions indicated by the dotted lines are shown for comparison of the ^27^Al lines hapes at different magnetic field strengths with associated Czjzek model^48^ line shape simulations (green lines). (d) Solid-state 1D ^27^Al MAS NMR spectrum of Al(OAr*)_3_ acquired at 28.2 T and 50 kHz MAS and static or MAS line shape simulations (green lines).

Interested in the ^27^Al spectroscopic signature of a true tricoordinate aluminum species with oxygen atoms in the first coordination sphere, we measured the tris(aryloxide) Al(OAr*)_3_ (Ar* = 2,6-di-*tert*-butyl-4-methyl-phenyl)^43^ as a model compound (Figure 3d). The compound exhibits a ^27^Al isotropic shift of 44 ppm and a quadrupolar coupling constant of 29.6 MHz, in very good agreement with values predicted from first-principles calculations (Table S4.1). The ^27^Al chemical shift of this compound is significantly shielded compared to what is expected for tricoordinate Al in aluminosilicates (δ_iso_ = 87 ppm and *C*_Q_ = 35 MHz),^44^ due to the differences of aryloxide vs (surface) siloxide ligands and associated σ/π effects,^45^ but shows similar quadrupolar coupling constants as expected from their similar trigonal planar geometry.^44^ At lower magnetic field strengths or without adequate MAS rates, static wide-line excitation and detection methods are necessary to extract the ^27^Al parameters of such Al sites,^46^ which would limit spectral resolution. Though such tricoordinate Al species have been proposed to exist under some conditions in aluminosilicate zeolites,^47^ their spectroscopic signatures have never been observed before and would be basically impossible to resolve at lower magnetic fields due to overlap of signals from other Al sites (Figure S4.1). Comparison of the ^27^Al spectra of mordenite zeolite and Al(OAr*)_3_ shows no evidence for large-*C*_Q_ species consistent with tricoordinate Al, corroborating our recent conclusion that the Lewis acid sites in mordenite zeolite under these conditions are predominantly pseudo-tricoordinate Al sites having a labile siloxane moiety coordinated.^41^

Overall, the adoption of high field (28.2 T) and fast MAS (>50 kHz) provides a substantial advantage for measurement of highly resolved solid-state NMR spectra of surfaces and materials. Though demonstrated only for select cases here, the methods will be extensible to diverse other inorganic, organometallic, and organic–inorganic hybrid materials. We anticipate that the advent and broader adoption of very high field NMR spectrometers and fast-spinning probe-heads including probes capable of MAS rates >100 kHz will additionally spur the development of new solid-state NMR pulse sequences, instrumentation, and methods to optimize sensitivity and resolution. In particular, these measurement conditions provide exceptional promise for high-resolution spectra of quadrupolar nuclei.

## Supporting Information Available

Experimental section, computational details, further characterization, additional solid-state NMR results and analyses (PDF)
