# Supplementary material for: Solid-State NMR Spectra of Protons and Quadrupolar Nuclei at 28.2 T: Resolving Signatures of Surface Sites with Fast Magic Angle Spinning
Source: JACS Au. 2022 Oct 25;2(11):2460–5. doi: 10.1021/jacsau.2c00510 (PMC9709951; doi:10.1021/jacsau.2c00510)
Supplement: Supplementary file 1 — au2c00510_si_001.pdf [file au2c00510_si_001.pdf]

Supporting information for:

Solid-state NMR spectra of protons and quadrupolar nuclei at 28.2 T: resolving signatures of surface sites with fast magic angle spinning

Zachariah J. Berkson,[†] Snædís Björgvinsdóttir,[†] Alexander Yakimov,[†] Domenico Gioffrè,[†] Maciej D. Korzyński [†‡] Alexander B. Barnes, [†] and Christophe Copéret[*†]*

[†]Department of Chemistry and Applied Biosciences, Vladimir Prelog Weg. 2, ETH Zürich, Zürich, Switzerland

* Christophe Copéret: ccoperet@ethz.ch

* Alexander B. Barnes: alexander.barnes@phys.chem.ethz.ch

‡ Present address (MDK): Department of Chemical and Physical Sciences, University of Toronto Mississauga, 3359 Mississauga Road Mississauga , Ontario L5L 1C6

Table of Contents

S1.1 Materials and general procedures 2
S1.2 Solid-state NMR analyses 2
Sample preparation and handling 2
Measurements at 28.2 T 3
Measurements at 16.4 T 3
S1.3 DFT calculations 4
S2. Well-defined silica-supported alkylidene 5
S3. Needle-shaped γ-alumina crystallites 8
S4. Spectroscopic signature of Al(III) in a tri-coordinate environment 13
S5. Additional references 14

S1. Experimental section

S1.1 Materials and general procedures

Unless otherwise stated, all materials were synthesized and handled under dry and oxygen-free argon atmosphere using Schlenk techniques or an MBraun or GS glovebox equipped with a purifier unit (<0.5 ppm O_2, <0.5 ppm H_2O). Syntheses were carried out using standard high vacuum lines (10^{-5} mbar) and glovebox techniques. All infrared (IR) spectra were recorded using a Bruker α-T spectrometer in an Ar glovebox equipped with OPUS software. A typical IR measurement consisted of acquisition of 32 scans in the region from 4000 to 400 cm^{-1}.

Mesoporous high surface area fumed silica (380 m^2/g, denoted hsa-SiO_2) was kindly provided by Evonik (EXP-4210-1) and was calcined at 500 °C under air for at least 4 h and treated under vacuum (10^{-5} mbar) at 500 °C for at least 12 h. Further thermal treatment under vacuum at 700 °C for at least 20 h (ramp rate 60 °C/h), yielded hsa-SiO_{2-700}. Titration of hsa-SiO_{2-700} using $BnMg_2$ yielded 0.51 mmol OH/g (0.9 OH/nm^2).

The W alkylidene (ArN)W(Me_2Pyr)$_2$(CHCMe_2Ph) (Ar = 3,5-dimethyl-phenyl; Me_2Pyr = 1,4-dimethylpyrrolide) was provided by XiMo. It was grafted on hsa-SiO_{2-700} following the procedure previously reported.[1]

Needle-shaped γ-alumina crystallites were synthesized as previously reported.[2]

Aluminosilicate mordenite zeolite was procured commercially in NH_4^+-form (Si/Al = 11, CBV21A, Zeolyst), calcined in an oven in static air at 550 °C for 6 h with a heating ramp rate of 1 °C min^{-1} and cooled down under ambient conditions. After calcination the samples were dehydrated at 450 °C under high vacuum (10^{-5} mbar) for 4 hours and stored in an Ar-filled glovebox.

The aluminum trisaryloxide Al(OAr*)$_3$ (Ar* = 2,6-tertbutyl-4-methyl-phenyl) was synthesized following the established literature procedure. [3] Lithium aluminum hydride (97%) was obtained from ABCR, 2,6-di-*tert*-butyl-4-methylphenol (>99.0%) was obtained from Sigma Aldrich, and toluene (≥99.8%) was purchased from Fisher Chemical. The solvent was dried using the commercial MBraun SPS-800 solvent purification system, degassed via three freeze-pump-thaw cycles, and stored over 4Å molecular sieves under argon.

S1.2 Solid-state NMR analyses

Sample preparation and handling

All materials were prepared for solid-state NMR analyses under dry and oxygen-free argon atmosphere in an MBraun or GS glovebox equipped with a purifier unit (<0.5 ppm O_2, <0.5 ppm H_2O). 1.3 mm or 3.2 mm zirconia NMR rotors were dried overnight at 150 °C and introduced into the glovebox while hot, and all plastic NMR rotor caps and packing tools were introduced into the glovebox overnight under vacuum to avoid contamination with air or water. Silica, alumina, or aluminosilicate materials were ground to a fine powder and carefully packed into the NMR rotors. The NMR rotors were capped with tight fitting Vespel plastic caps and sealed under argon in vials

with screw-cap tops and silicone/PTFE gaskets, which were further sealed with electrical tape. The NMR rotors were transferred under Ar to the NMR spectrometer, rapidly inserted into the MAS NMR probe, and spun with dry N_2 gas.

For the measurements using 1.3 mm rotors, samples were typically rotated at the maximum accessible MAS rate with acceptable spinning stability; 50-65 kHz MAS depending on the sample, cap, and rotor packing. Optimal spinning stability (typically +/- 5 Hz or less) was achieved using pristine Vespel plastic drive caps; the integrity of the drive cap was checked via optical microscope before inserting the rotors into the NMR probehead.

^{1}H chemical shifts were referenced to tetramethylsilane at 0.0 ppm using the isolated Si-OH peak of dehydroxylated silica (1.7 ppm) as an external secondary reference. ^{27}Al shifts were referenced to a 1 M aqueous solution of $Al(NO_3)_3$ at 0 ppm.

All ^{1}H-detected 2D NMR spectra were acquired with ^{1}H presaturation consisting of 30 $\pi/2$ pulses separated by 0.197 ms delays.

All lineshape simulations were performed using DMFIT software.[4]

Measurements at 28.2 T

1D and 2D solid-state ^{1}H and ^{27}Al MAS NMR spectra were acquired on a 1200 MHz (28.2 T) Bruker Avance NEO spectrometer equipped with a broadband 1.3 mm HXY MAS probehead and operating at Larmor frequencies of 1200.956 and 312.943 MHz for ^{1}H and ^{27}Al, respectively. Rotor spinning rates were controlled using a Bruker MAS III unit. The temperature measured in the gas stream close to the NMR rotor was maintained at 285 K using a Bruker VTU unit. The ^{1}H and ^{27}Al spectra were acquired with 130 kHz (1.9 μs) or 156 kHz (1.6 μs) rf field amplitude, respectively. All single pulse 1D ^{27}Al MAS NMR spectra were acquired using $\pi/12$ tip angle. Typically, relaxation delays for ^{1}H detected experiments were set to 3 s or 1.3x the ^{1}H spin-lattice relaxation time as measured by ^{1}H saturation recovery. The 2D ^{1}H{^{27}Al} correlation spectra of needle-shaped gamma alumina crystallites and mordenite zeolite were acquired using a 2D arbitrary indirect dwell time[5] (AID) dipolar-mediated heteronuclear multiple quantum coherence (D-HMQC) sequence with 12 rotor periods of SR4 recoupling[6] to reintroduce the ^{1}H-^{27}Al dipole-dipole couplings. ^{1}H spin-spin T_2 relaxation times were measured using a pseudo-2D CPMG sequence[7] with varied lengths (τ) of rotor-synchronized echo trains. All 1D ^{1}H echo MAS spectra were acquired using $\pi/2$-τ- π-τ echo sequences with τ typically equal to 10 rotor periods.

Measurements at 16.4 T

1D and 2D solid-state ^{1}H and ^{27}Al MAS NMR spectra were acquired on a 700 MHz (16.4 T) Bruker Avance NEO spectrometer operating at Larmor frequencies of 700.132 and 182.438 MHz for ^{1}H and ^{27}Al, respectively. Spectra were measured either at 16-20 kHz MAS using a broadband 3.2 mm HX MAS probe or at 40-50 kHz MAS using a broadband 1.3 mm HX MAS probe. The 2D ^{1}H{^{27}Al} AID-D-HMQC and ^{27}Al triple-quantum (TQ) MAS spectra were acquired under analogous conditions to the measurements conducted at 28.2 T.

S1.3 DFT calculations

^{27}Al NMR calculations

The geometry of $Al(OAr^*)_3$ (Ar* = 2,6-di-tert-butyl-4-methyl-phenyl) was optimized using Gaussian 09 starting from the reported crystal structure.[3] The geometry optimization was performed using the B3LYP[8] functional in combination with Pople's 6-31G(d) basis sets.[9] NMR calculations were performed using ADF 2019 with the PBE0 functional and TZ2P Slater-type basis sets.[10] Relativistic effects were treated by the 2-component zeroth order regular approximation (ZORA).[11–13] Calculated ^{27}Al chemical shifts were referenced to $Al(acac)_3$ (acac = acetylacetonate), with an experimental isotropic chemical shift of 0 ppm and a calculated isotropic shielding of 590.4.

S2. Well-defined silica-supported alkylidene

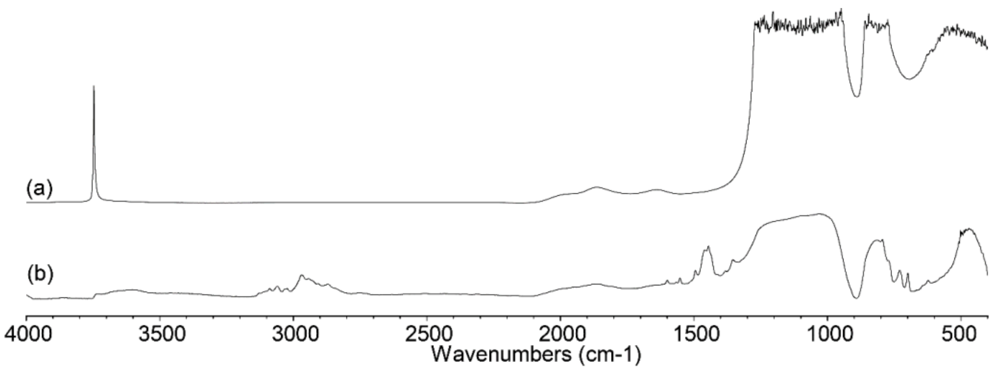

Figure S2.1. Transmission FTIR spectra of (a) hsa-$SiO_{2\text{-}700}$ and (b) W alkylidene supported on hsa-$SiO_{2\text{-}700}$. All spectra are normalized to the intensity of signals from siloxane bridge vibrations in the 1500-2100 cm^{-1} region.

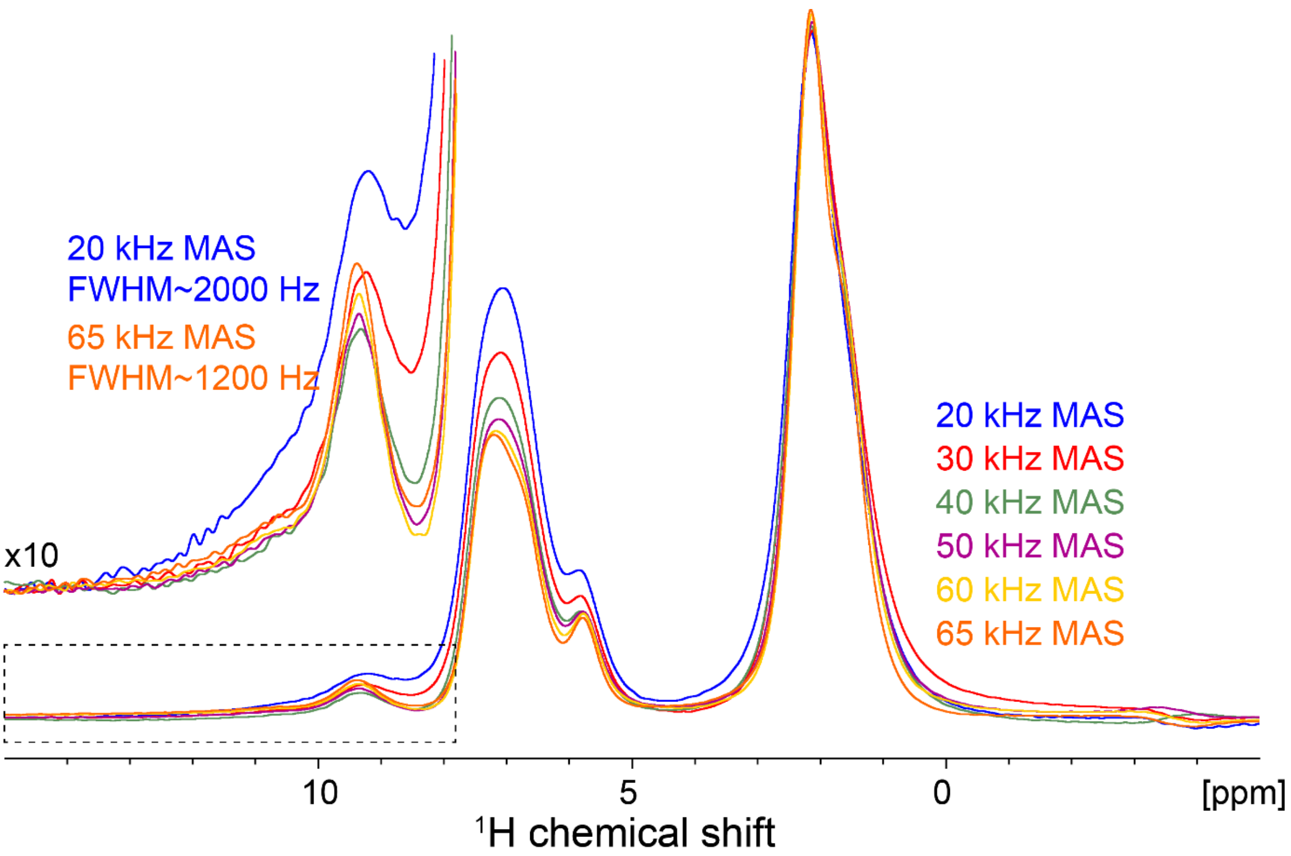

Figure S2.2. Solid-state 1D 1H echo MAS NMR spectra of silica-supported W alkylidene acquired at 20-65 kHz MAS, 28.2 T, and 285 K.

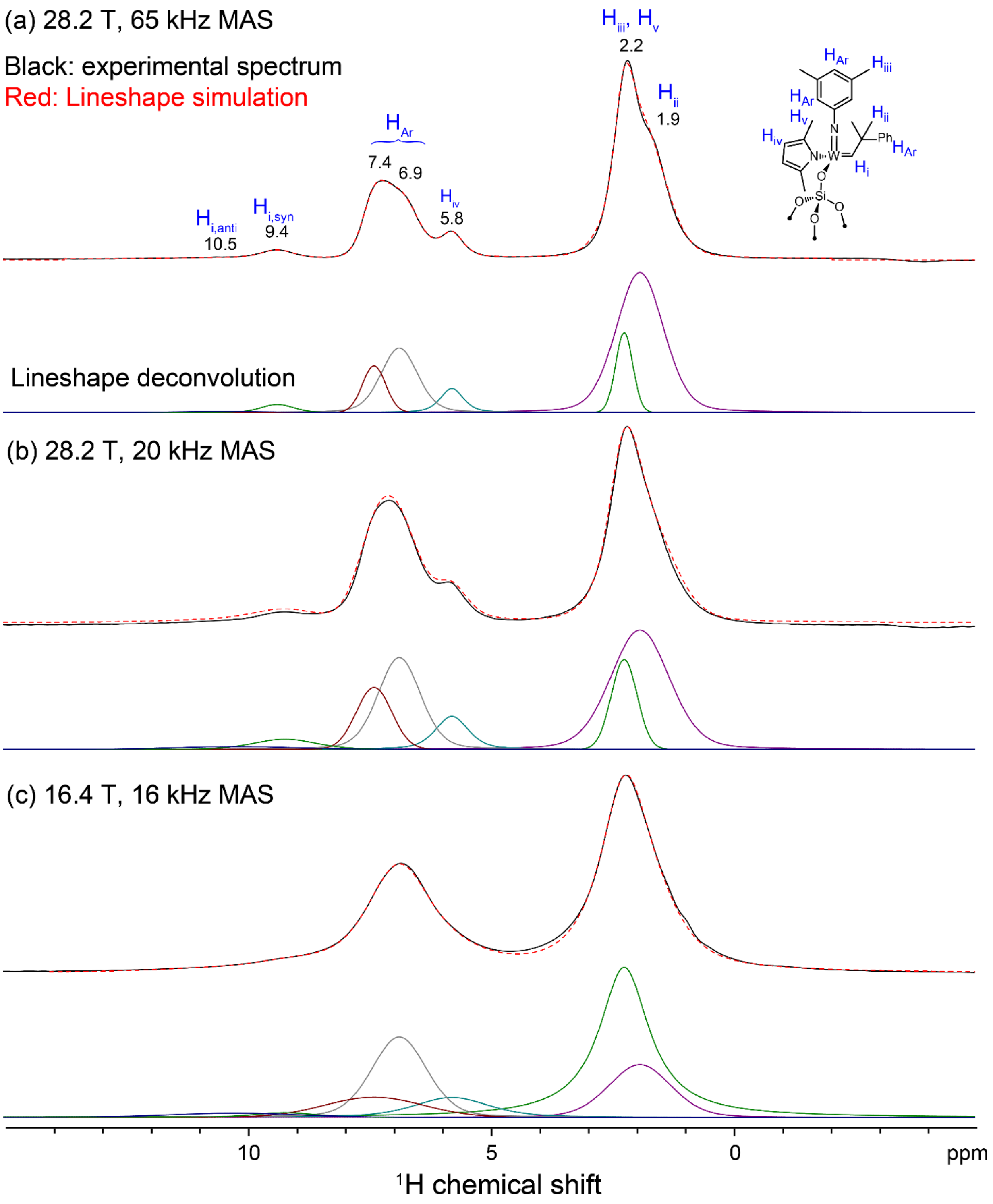

Figure S2.3. Solid-state 1D 1H echo MAS NMR spectra (black), simulated lineshapes (red), and lineshape deconvolutions of silica-supported W alkylidene for spectra acquired at 28.2 T and (a) 65 kHz MAS or (b) 20 kHz MAS) or (c) 16.4 T and 16 kHz MAS (same spectra as Figure 1 in the main text). Fit parameters are provided in Table S2.1.

Table S2.1. ^{1}H Fit parameters for the spectral deconvolutions shown in Figure 2.3. Mixed Gaussian/Lorentzian lineshapes were used, with peak positions fixed allowing linewidths to vary. Note that the integrated intensities are non-quantitative due to differences in relaxation parameters for the different ^{1}H signals.

Signal	δ_{iso} (ppm)	Assignment	Linewidth (ppm)			% intensity		
			65 kHz/ 28.2 T	20 kHz/ 28.2 T	16 kHz/ 16.4 T	65 kHz/ 28.2 T	20 kHz/ 28.2 T	16 kHz /16.4 T
1	1.9	H_{ii}	1.2	1.4	1.5	54	41	15
2	2.2	H_{iii}, H_{V}	0.4	0.6	1.2	10	13	46
3	5.8	H_{iv}	0.6	0.8	1.8	5	7	7
4	6.9	H_{Ar}	0.9	1.0	1.3	20	23	20
5	7.4	H_{Ar}	0.6	0.9	2.4	8	12	8
6	9.4	$H_{i,syn}$	0.8	1.5	1.5*	2	4	1
7	10.5	$H_{i,anti}$	1.9	3.0*	3.0*	1	2	2

*Not resolved

S3. Needle-shaped γ-alumina crystallites

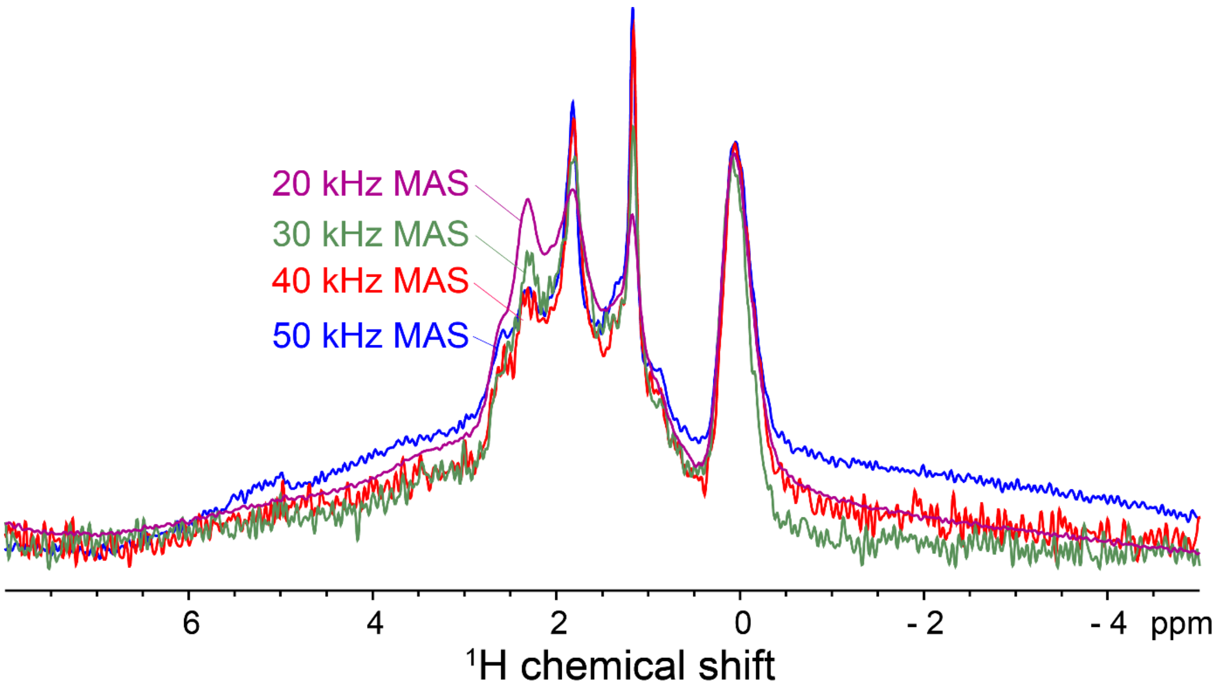

Figure S3.1. Solid-state 1D 1H echo MAS NMR spectra of needle-shaped γ-alumina crystallites acquired at 20-50 kHz MAS, 28.2 T, and 285 K.

Table S3.1. Results of ^{1}H T_1 and T_2 relaxation time analyses of needle-shaped γ-alumina crystallites

^{1}H shift (ppm)	Signal assignment[2,14]	$T_{1,long}$ (s)[a]	$T_{1,short}$ (s)[a]	Fraction of slow-relaxing species[a,b]	T_2 (ms)[c]
-0.1	μ_1-OH	28	2.6	0.79	190
1.1	μ_2-OH	52	1.8	0.53	1.8
1.7	μ_2-OH	13	0.001	0.94	1.5
2.2	μ_2-OH	59	0.7	0.93	0.7
2.5	μ_2-OH	92	3.6	0.84	0.4
3.2	H-bond donors	210	2.5	0.90	0.2

[a] Extracted from saturation-recovery data using a bi-exponential fitting function of the form $I(\tau) = I_{0,long}(1 - e^{(T_{1,long}/\tau)}) + I_{0,short}(1 - e^{(T_{1,short}/\tau)})$ where I is the signal intensity (separated into slow- and fast-relaxing components) and τ is the relaxation delay time.

[b] $I_{0,long}/(I_{0,long} + I_{0,short})$

[c] Extracted from CPMG data using a decaying single exponential fitting function.

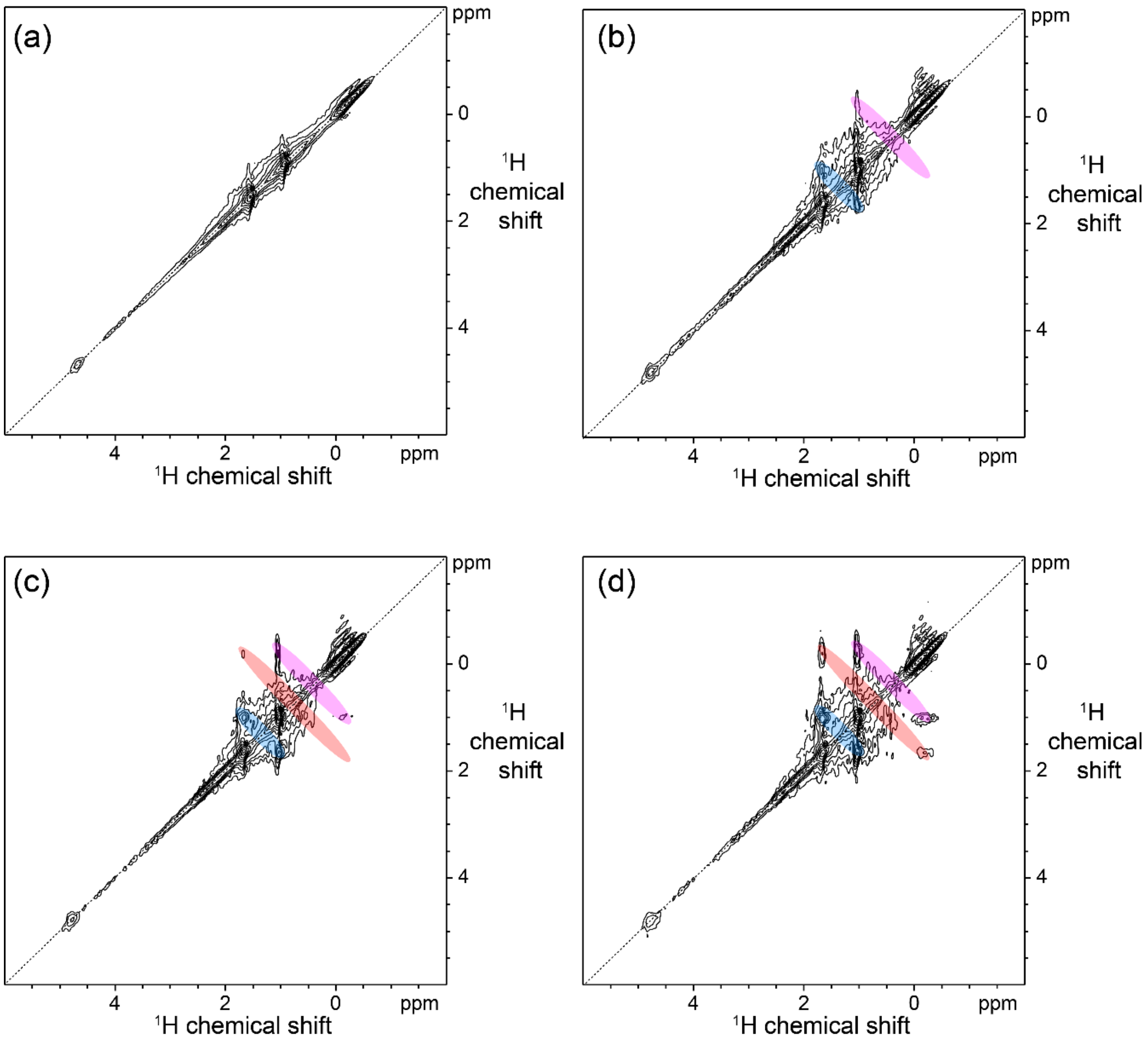

Figure S3.2. Solid-state 2D $^{1}H\{^{1}H\}$ NOE correlation spectroscopy of needle-shaped γ-alumina crystallites with mixing times of (a) 3 μs, (b) 25 ms, (c) 75 ms, and (d) 200 ms. Dotted lines indicate the 1:1 diagonal. Shaded regions indicate off-diagonal correlations that arise from mutually proximate ^{1}H moieties interacting through chemical or dipolar exchange over the mixing period.[15] At short mixing times (25 ms) the ^{1}H signals at 1.7 and 2.5 ppm are prominently correlated (blue shaded region), indicating that the corresponding μ_2-OH species are in relatively close nanoscale proximity, possibly occupying the same crystal facet. The ^{1}H signal at 1.7 ppm is also weakly correlated to the ^{1}H signal at -0.1 ppm (pink shaded region) from μ_1-OH species that are thought to occupy edge sites,[2,14] suggesting that only a single spectroscopically distinct μ_2-OH species is close to the edges of the crystal facets. At longer mixing times both these signals increase in intensity, and a new correlation emerges between the ^{1}H signals at 2.5 and -0.1 ppm (red-shaded region) indicating that the μ_2-OH species associated with the ^{1}H signal at 2.5 ppm are further from the crystallite edges, but still close enough for slow chemical and/or dipolar exchange processes. The absence of correlated off-diagonal signal intensity associated with signals from H bond donors (> 2.5 ppm) shows that these species are spatially segregated from the other surface OH groups.

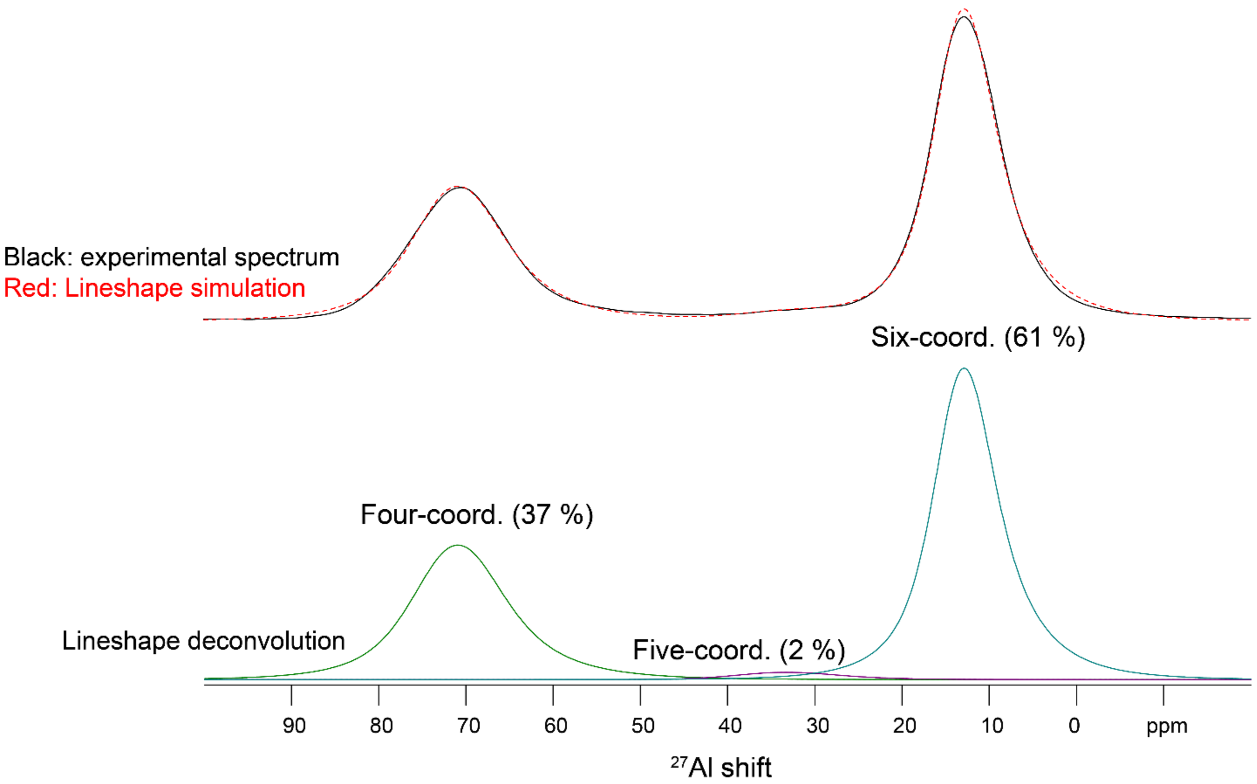

Figure S3.3. Solid-state quantitative 1D single-pulse ^{27}Al MAS NMR spectrum of needle-shaped γ-alumina crystallites acquired at 60 kHz MAS, 28.2 T, 285 K, with a relaxation delay of 0.5 s, and a π/12 tip angle.

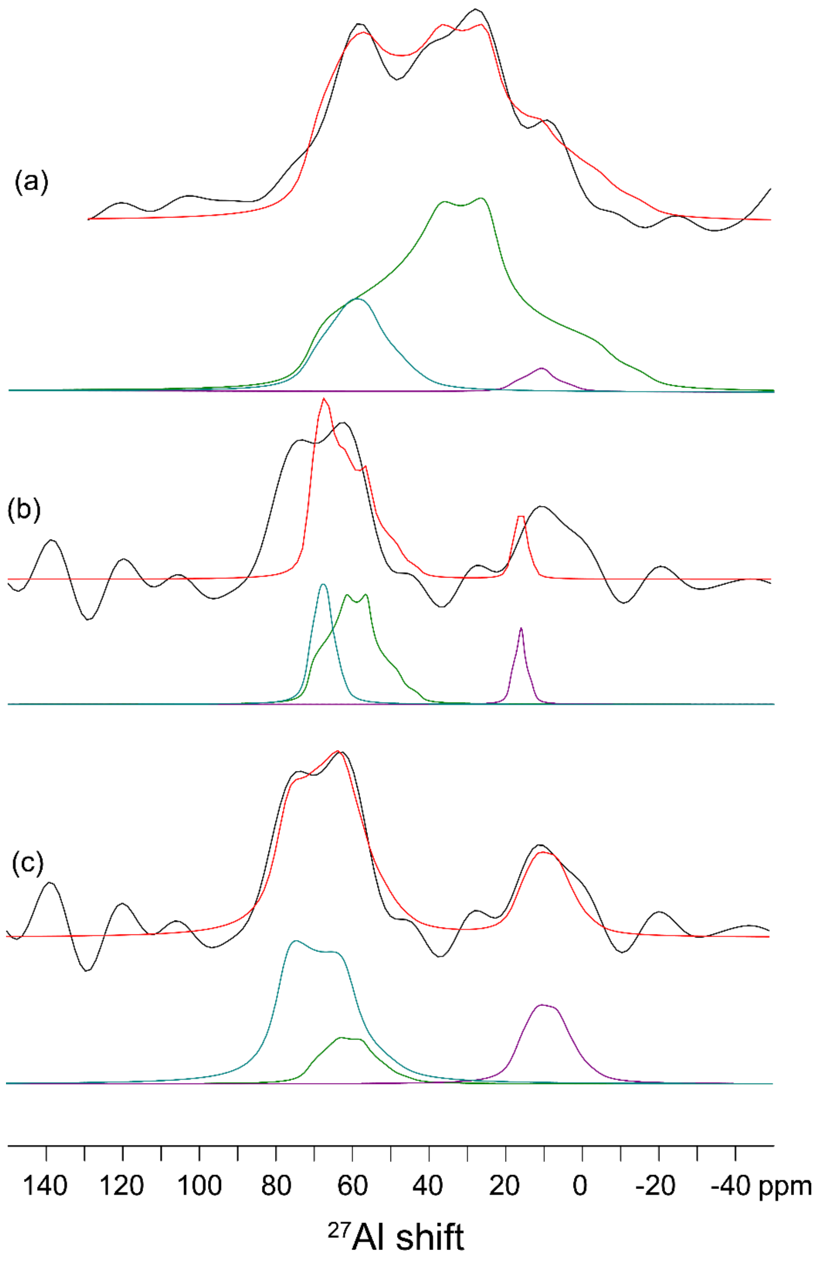

Figure S3.4. 1D slices through the ^{27}Al dimension (black) and lineshape simulations (red) of the 2D ^{1}H{^{27}Al} AID-D-HMQC spectra of needle-shaped γ-alumina crystallites acquired at (a) 16.4 T, 20 kHz MAS or (b,c) 28.2 T, 50 kHz MAS. The slices were extracted from the spectra shown in Figure 2d in the main text at ^{1}H positions of -0.1 ppm. The lineshapes in (a) and (b) were simulated using the ^{27}Al parameters recently reported from 2D measurements with varying recoupling delays at 16.4 T,[2] showing that these parameters account for the lineshape observed at lower magnetic field strengths but cannot account for the lineshape measured at high fields and fast-MAS. The simulated lineshape in (c) was fitted allowing chemical shift and C_Q values to freely vary, providing a much better match to the experimental data and evidence for signals with larger C_Qs consistent with values expected for surface sites.[16] Simulated lineshape parameters are provided in Table S3.2.

Table S3.2. Lineshape analyses of ^{27}Al slices extracted from the 2D $^{1}H\{^{27}Al\}$ AID-D-HMQC spectra of needle-shaped γ-alumina crystallites (Figure 2 in the main text)[a]

Component	Signal assignment	δ_{iso} (ppm)	C_Q (MHz)	η[b]
16.4 T[c]				
1	Al_{IV}	72	8.0	0.7
2	Al_{IV}	72	13.5	0.7
3	Al_{VI}	19	6.0	0.8
28.2 T				
1	Al_{IV}	82	15.5	0.1
2	Al_{IV}	72	13.2	0.5
3	Al_{VI}	18	11.8	0.5

[a] Extracted at ^{1}H positions of -0.1 ppm.

[b] Asymmetry parameter; allowed to freely vary to obtain best fits to the experimental data

[c] Values reported in Ref. 2

S4. Spectroscopic signature of Al(III) in a tri-coordinate environment

Table S4.1. DFT calculated ^{27}Al NMR parameters of Al(OAr*)$_3$ (Ar* = 2,6-di-*tert*-butyl-4-methyl-phenyl) and Al(acac)$_3$ (acac = acetylacetonate) as a chemical shift reference

Compound	δ_{iso} (ppm)	C_Q (MHz)	η
Al(acac)$_3$ (calculated)	0	2.7	0.01
Al(OAr*)$_3$ (calculated)	40	28.3	0.02
Al(OAr*)$_3$ (Experimental)	44	29.6	0.05

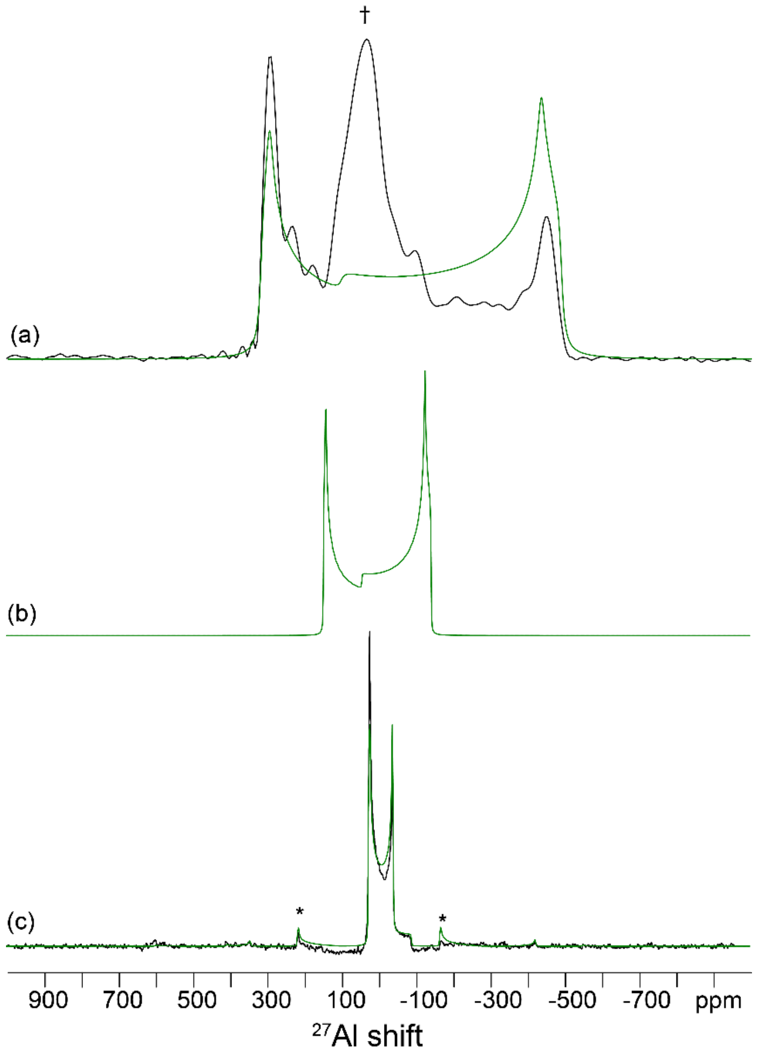

Figure S4.1. Comparison of solid-state ^{27}Al NMR spectra of Al(OAr*)$_3$: (a) 1D ^{27}Al WURST-QCPMG[17] spectrum acquired at 16.4 T and static conditions (black), along with lineshape simulation (green), (b) lineshape simulation at 28.2 T and static conditions, and (c) 1D ^{27}Al echo MAS NMR spectrum acquired at 28.2 T and 60 kHz MAS (black), along with lineshape simulation (green); same data as shown in Fig. 3d of the main text. * indicates spinning sidebands, † indicates an alumina background signal.

S5. Additional references

(1) Mougel, V.; Santiago, C. B.; Zhizhko, P. A.; Bess, E. N.; Varga, J.; Frater, G.; Sigman, M. S.; Copéret, C. Quantitatively Analyzing Metathesis Catalyst Activity and Structural Features in Silica-Supported Tungsten Imido-Alkylidene Complexes. *J. Am. Chem. Soc.* **2015**, *137* (20), 6699–6704.

(2) Völker, L. A.; Meyet, J.; Berkson, Z. J.; Rochlitz, L.; Van Bokhoven, J. A.; Copéret, C. Revisiting Edge Sites of γ-Al2O3 Using Needle-Shaped Nanocrystals and Recoupling-Time-Encoded {27Al}-1H D-HMQC NMR Spectroscopy. *J. Phys. Chem. C* **2022**, *126* (14), 6351–6360.

(3) Healy, M. D.; Barron, A. R. Synthesis and Structure of Al(OAr*)3 (Ar* = 2,6-tBu_2-4-MeC_6H_2): The First Three-Coordinate Homoleptic Aluminum Aryloxide. *Angew. Chemie Int. Ed. English* **1992**, *31* (7), 921–922.

(4) Massiot, D.; Fayon, F.; Capron, M.; King, I.; Le Calvé, S.; Alonso, B.; Durand, J. O.; Bujoli, B.; Gan, Z.; Hoatson, G. Modelling One- and Two-Dimensional Solid-State NMR Spectra. *Magn. Reson. Chem.* **2002**, *40* (1), 70–76.

(5) Venkatesh, A.; Perras, F. A.; Rossini, A. J. Proton-Detected Solid-State NMR Spectroscopy of Spin-1/2 Nuclei with Large Chemical Shift Anisotropy. *J. Magn. Reson.* **2021**, *327*, 106983.

(6) Brinkmann, A.; Kentgens, A. P. M. Proton-Selective O-17-H-1 Distance Measurements in Fast Magic-Angle-Spinning Solid-State NMR Spectroscopy for the Determination of Hydrogen Bond Lengths. *J. Am. Chem. Soc.* **2006**, *128* (46), 14758–14759.

(7) Meiboom, S.; Gill, D. Modified Spin-Echo Method for Measuring Nuclear Relaxation Times. *Rev. Sci. Instrum.* **1958**, *29* (8), 688–691.

(8) Yanai, T.; Tew, D. P.; Handy, N. C. A New Hybrid Exchange-Correlation Functional Using the Coulomb-Attenuating Method (CAM-B3LYP). *Chem. Phys. Lett.* **2004**, *393* (1–3), 51–57.

(9) Rassolov, V. A.; Pople, J. A.; Ratner, M. A.; Windus, T. L. 6-31G* Basis Set for Atoms K through Zn. *J. Chem. Phys.* **1998**, *109* (4), 1223–1229.

(10) Velde, G.; Bickelhaupt, F. M.; van Gisbergen, S. J. A.; Guerra, C. F.; Baerends, E. J.; Snijders, J. G.; Ziegler, T. Chemsitry with ADF. *J. Comput. Chem.* **2001**, *22* (9), 931.

(11) Lenthe, E. van; Baerends, E. J.; Snijders, J. G. Relativistic Regular Two-component Hamiltonians. *J. Chem. Phys.* **1993**, *99* (6), 4597–4610.

(12) Van Lenthe, E.; Snijders, J. G.; Baerends, E. J. The Zero-Order Regular Approximation for Relativistic Effects: The Effect of Spin-Orbit Coupling in Closed Shell Molecules. *J. Chem. Phys.* **1996**, *105* (15), 6505–6516.

(13) Autschbach, J. The Role of the Exchange-Correlation Response Kernel and Scaling Corrections in Relativistic Density Functional Nuclear Magnetic Shielding Calculations

with the Zeroth-Order Regular Approximation. *Mol. Phys.* **2013**, *111* (16–17), 2544–2554.

(14) Batista, A. T. F.; Wisser, D.; Pigeon, T.; Gajan, D.; Diehl, F.; Rivallan, M.; Catita, L.; Gay, A. S.; Lesage, A.; Chizallet, C.; et al. Beyond Γ-Al2O3 Crystallite Surfaces: The Hidden Features of Edges Revealed by Solid-State 1H NMR and DFT Calculations. *J. Catal.* **2019**, *378*, 140–143.

(15) Mroue, K. H.; Nishiyama, Y.; Kumar Pandey, M.; Gong, B.; McNerny, E.; Kohn, D. H.; Morris, M. D.; Ramamoorthy, A. Proton-Detected Solid-State NMR Spectroscopy of Bone with Ultrafast Magic Angle Spinning. *Sci. Rep.* **2015**, *5*, 1–10.

(16) Wischert, R.; Florian, P.; Copéret, C.; Massiot, D.; Sautet, P. Visibility of Al Surface Sites of γ-Alumina: A Combined Computational and Experimental Point of View. *J. Phys. Chem. C* **2014**, *118* (28), 15292–15299.

(17) Schurko, R. W. Ultra-Wideline Solid-State NMR Spectroscopy. *Acc. Chem. Res.* **2013**, *46* (9), 1985–1995.
